# Severe Necrotizing Fasciitis Following Minor Neglected Left-Hand Wound Complicated With Toxic Shock Syndrome and Multiorgan Failure: A Case Report and Literature Review

**DOI:** 10.7759/cureus.90537

**Published:** 2025-08-19

**Authors:** Ahmad Hassanien, Khaled Sewify, Abdulaziz Alshaer, Wael Gomaa, Mohamed Shararibo

**Affiliations:** 1 Critical Care, King Fahad Military Medical Complex, Dhahran, SAU

**Keywords:** group a streptococcus, intravenous immunoglobulins., multiorgan dysfunction, surgical debridement, toxic shock syndrome

## Abstract

Necrotizing fasciitis (NF) is a life-threatening soft tissue infection requiring urgent intervention. Despite advances in care, delays in diagnosis contribute to high morbidity and mortality. Here, we present a 17-year-old male patient previously healthy, who developed NF and toxic shock syndrome (TSS) following minor hand trauma. He developed multiorgan failure in the form of acute respiratory distress (ARDS), circulatory failure, high hepatic enzymes, and coagulopathy. The patient was managed successfully with early surgical debridement, antibiotics, immunoglobulins, and critical care support, including invasive mechanical ventilation and high-dose vasopressors and inotropic support. This case highlights NF’s rapid progression in healthy individuals and the necessity of aggressive multidisciplinary management. Early recognition, prompt surgery, and tailored therapies remain critical. NF demands high clinical suspicion even in low-risk populations. Aggressive intervention and adherence to guidelines improve survival and functional outcomes.

## Introduction

Necrotizing fasciitis (NF) represents a severe, rapidly advancing infection affecting soft tissues, resulting in widespread fascial and subcutaneous necrosis, with reported mortality between 10% and 20% [1]. Historical accounts trace its recognition to Hippocrates in ancient Greece, with formal nomenclature established by Joseph Jones in 1871, yet this condition continues to challenge modern clinicians [1].

Although traditionally associated with immunocompromised patients, contemporary literature increasingly documents severe cases in previously healthy individuals. Invasive Group A *Streptococcus *(GAS) infections causing NF, frequently complicated by streptococcal toxic shock syndrome (STSS), are responsible for 40% of deaths from invasive GAS disease [2].

Current epidemiological data reveal NSTI incidence of 3.8-10.3 per 100,000 individuals yearly in industrialized nations, maintaining mortality rates of 10-20% even with modern treatment approaches [2]. Contemporary epidemiological research indicates a global increase in these infections, potentially linked to growing rates of diabetes, obesity, and immunosuppressive therapies [3].

Necrotizing soft tissue infections (NSTIs) are categorized based on microbial etiology into polymicrobial (type 1) and monomicrobial (type 2) forms [4]. Type 1 infections, comprising 70-80% of NSTIs, predominantly affect elderly patients with significant comorbidities [4]. Type 2 infections, primarily due to GAS or *Staphylococcus aureus*, may affect healthy persons across all age groups [4]. Further categories encompass type 3 (marine pathogen-related, particularly *Vibrio *species) and type 4 (fungal origin) [4].

The disease mechanism in GAS-related NF involves multiple bacterial virulence determinants, notably M proteins, streptolysins, and superantigens, which enable tissue invasion while triggering overwhelming inflammatory responses [5]. STSS develops in approximately 40% of GAS NF cases, manifesting as profound systemic toxicity, circulatory collapse, and multiple organ dysfunction [5].

Diagnosing NF presents significant challenges, as initial presentations frequently mimic simple cellulitis, leading to misdiagnosis in 41-96% of cases at first evaluation [6]. The Laboratory Risk Indicator for Necrotizing Fasciitis (LRINEC) scoring system was created to facilitate early detection, demonstrating a sensitivity of 68% and specificity of 84% [7]. Nevertheless, clinical acumen remains essential given the inherent limitations of scoring tools [7].

Current management strategies prioritize immediate aggressive surgical intervention, targeted antimicrobial coverage, and comprehensive supportive measures. Multiple investigations confirm superior outcomes with early surgical debridement, as mortality rises substantially when surgery is postponed beyond 24 hours [8]. Supplementary treatments such as hyperbaric oxygen and immunoglobulin therapy demonstrate potential benefits in specific cases, especially those complicated by STSS [9]. We describe our experience managing a young, previously healthy patient who developed GAS NF complicated by STSS, highlighting diagnostic challenges and treatment approaches employed at our institution.

## Case presentation

A previously healthy 17-year-old male college student was transferred to our facility (KFMMC, Dhahran) for advanced management of a rapidly progressing left-hand infection. One week prior, a minor hand wound, exacerbated by exercise, was initially diagnosed as cellulitis at a regional hospital.

**Figure 1:**
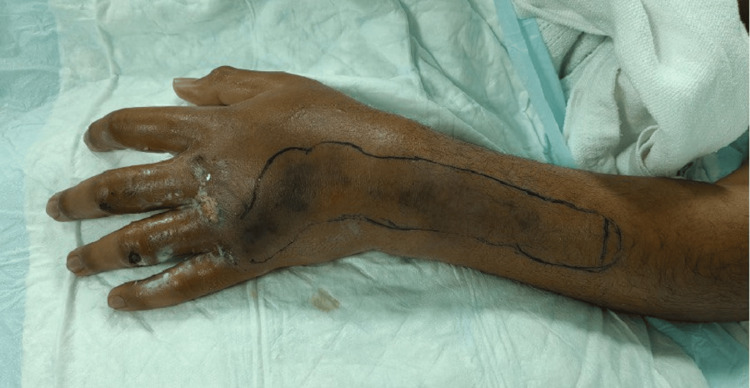
The left upper limb upon admission The image demonstrates dusky discoloration, significant edema, and compromised perfusion of the left hand and forearm characteristic of necrotizing fasciitis. Note the violaceous skin changes and marked swelling

Shortly after starting broad-spectrum antibiotics (piperacillin/tazobactam), he developed septic shock and respiratory failure requiring transfer. Notably, five other students from the same group reported similar symptoms. Initial labs revealed sepsis, liver injury, hypoperfusion, and hypoxia. On arrival, he was drowsy, cyanotic, and in respiratory distress despite noninvasive ventilation (NIV). His left hand showed dusky, discolored skin, significant swelling, and impaired movement. The pulse was palpable; however, the capillary refill time was prolonged in comparison to the right hand (Figure 1).

Subsequent workup revealed leucocytosis 43,000, coagulopathy with an international normalized ratio (INR) of 2.3, partial thromboplastin time (PTT) of 46, and elevated liver enzymes with alanine aminotransferase (ALT) of 123, aspartate aminotransferase (AST) of 132, procalcitonin recorded at 26.4, and C-reactive protein (CRP) at 293 and full septic screen done. The LRINEC score was markedly elevated at 6, thereby indicating a significant suspicion of NF. Ultrasound-Doppler examination of the left upper limb demonstrated diffuse subcutaneous edema in the left hand, with no evidence of venous thromboembolism (VTE) or localized fluid collection.

**Figure 2:**
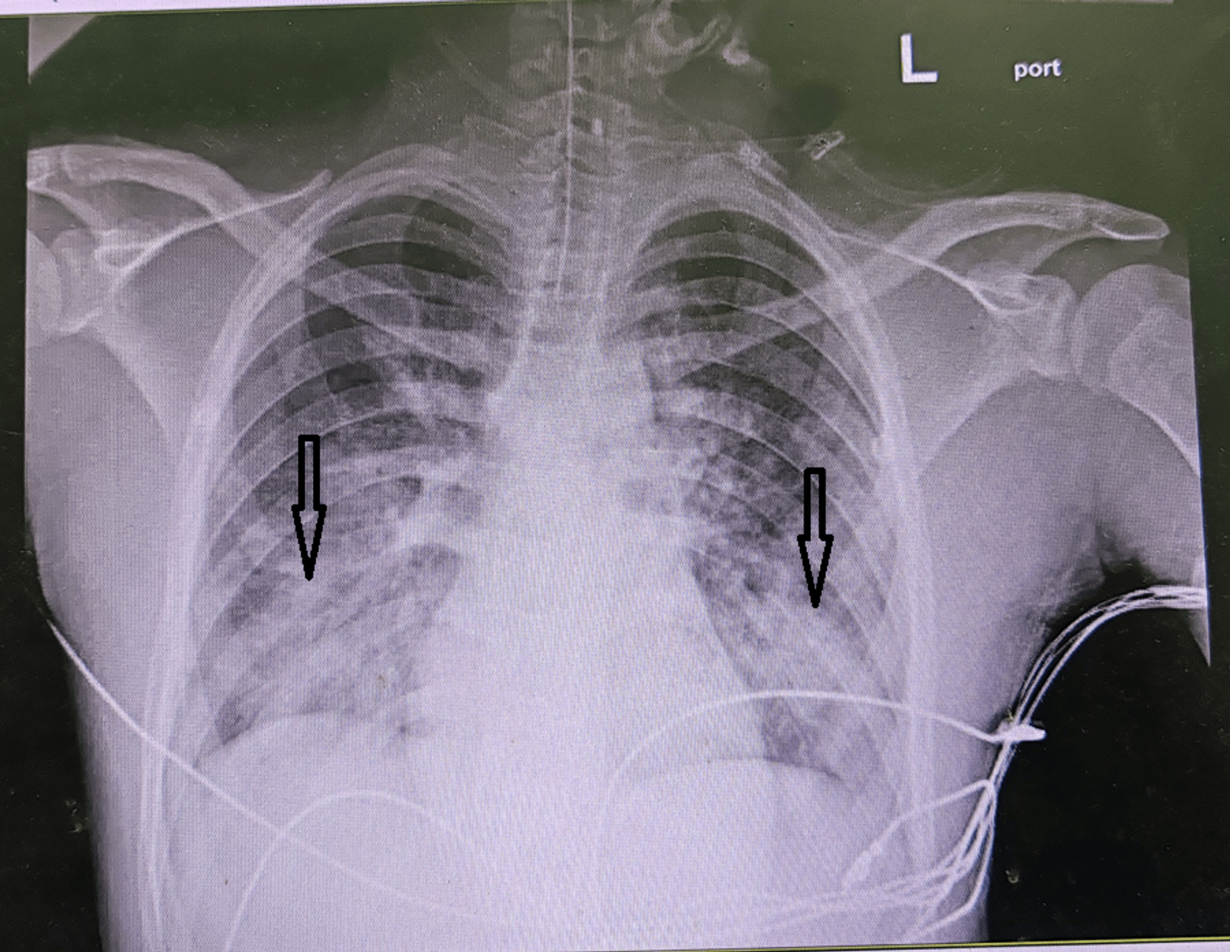
Chest X-ray on admission Bilateral patchy infiltrates (indicated by arrows) consistent with acute respiratory distress syndrome (ARDS) secondary to sepsis. The infiltrates are predominantly in the lower lung fields bilaterally, with preserved lung volumes

Echocardiography indicated a reduction in left ventricular function, with an ejection fraction (EF) of 35%. He was intubated, mechanically ventilated, started on intravenous immunoglobulin (IVIG), and underwent urgent fasciotomy and debridement. Chest X-ray (CXR) demonstrated bilateral patchy infiltrates as shown in Figure 2.

Management

**Figure 3:**
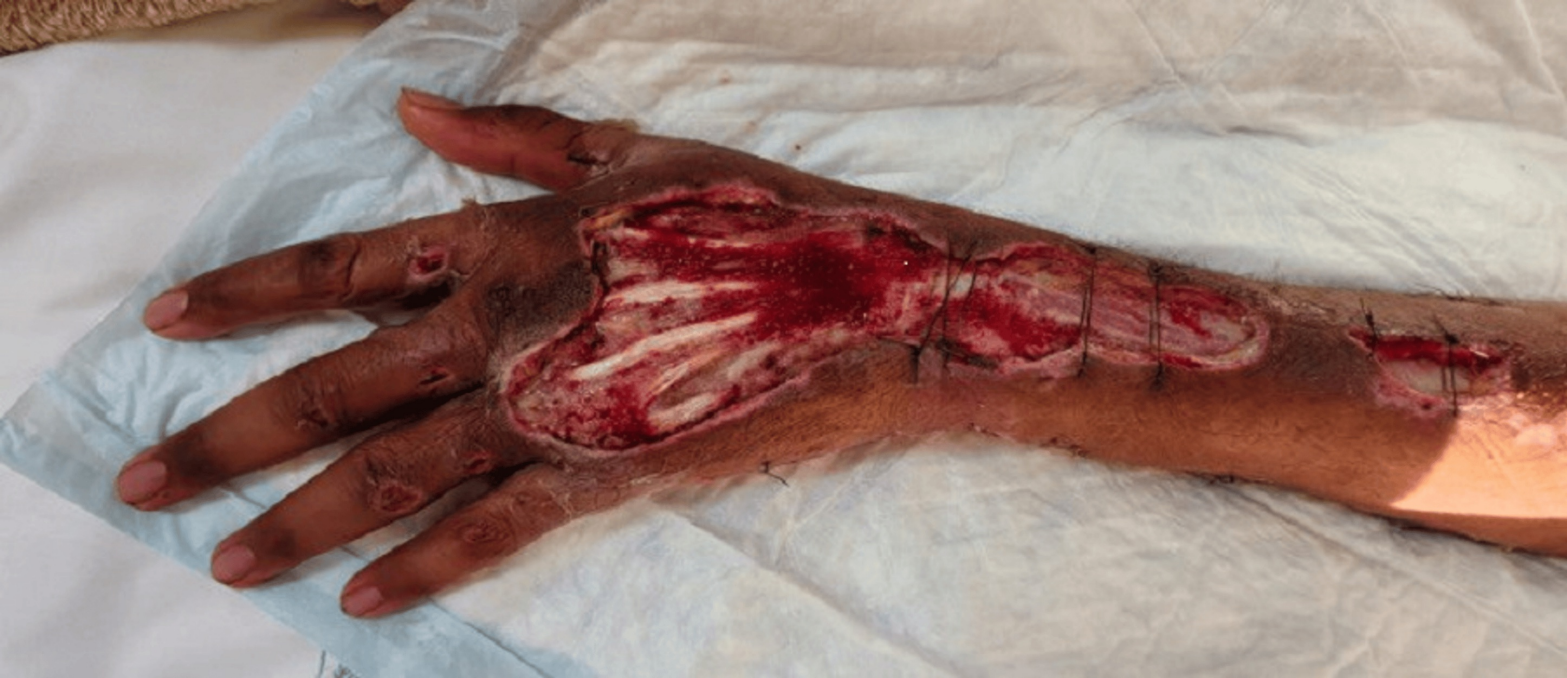
The left upper limb after fasciotomy and multiple debridements The image shows the left hand and forearm after fasciotomy and multiple surgical debridements. Healthy granulation tissue is visible with marked improvement in tissue viability compared to the initial presentation. The wound edges show signs of healing with no evidence of ongoing necrosis

On admission, the patient was intubated and mechanically ventilated for severe acute respiratory distress (ARDS), adhering to ARDSnet protocols. Fluid resuscitation was started along with noradrenaline infusion with an initial dose ranging between 0.2 and 0.3 mcg/kg/min. Antibiotics were broadened to linezolid, amikacin, and piperacillin/tazobactam, and IVIG (1 g/kg) was administered over two days. Urgent surgical fasciotomy and debridement were performed; tissue cultures were obtained. On day 2, prone positioning was initiated for persistent hypoxia (16 hours/day for three days). Milrinone was added based on echocardiography. Daily wound debridement and washing were continued for five days, and the wound showed remarkable improvement as revealed in Figure 3. Wound cultures grew *Streptococcus pyogenes*. Antibiotics were de-escalated from linezolid to ampicillin and continued for a total of 14 days. Follow-up wound cultures showed no growth.

**Figure 4:**
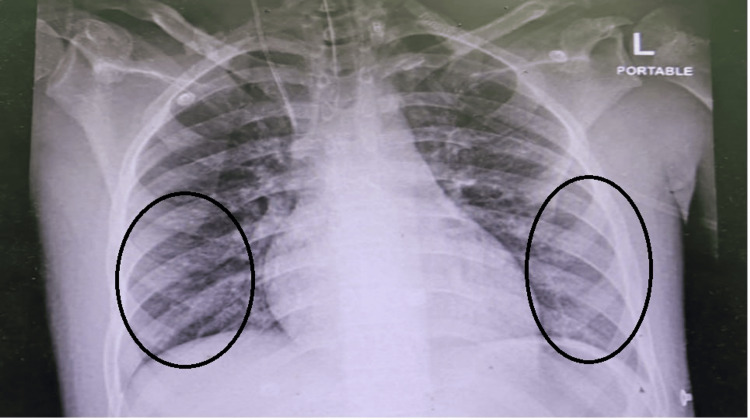
The X-ray chest revealed marked improvement Marked improvement in bilateral infiltrates with near-complete resolution of the previously noted opacities (circled areas show regions of improvement). The lung fields appear clear with a normal cardiomediastinal silhouette

On day 6, the patient had remarkably improved and was hemodynamically stable, weaned off vasopressors and inotropes. The oxygen requirement (P/F ratio improved above 300 from 97 on admission), and CXR significantly improved, and the patient was successfully extubated (Figure 4).

Two days after extubation, the patient was shifted to the surgical ward under the care of plastic surgery, and the antibiotic course continued for a total of 10 days. Table 1 reveals lab results during the patient's stay in the ICU.

**Table 1:** The laboratory results from (day 1-6) WBC: white blood cell; CRP; C-reactive protein; INR: international normalized ratio; PTT: partial thromboplastin time; AST: aspartate aminotransferase; ALT: alanine aminotransferase; BUN; blood urea nitrogen

Parameter	Day 1	Day 3	Day 6	Reference range
WBC count (×10³/µL)	43.3	18	12	4.5-11.0
Procalcitonin (ng/mL)	26.4	2	0.28	<0.5
CRP (mg/L)	293	247	79	<10
INR	2.3	1.07	1.06	0.8-1.2
PTT (seconds)	46	35	39	25-35
AST (IU/L)	81	52	51	10-40
ALT (IU/L)	89	42	62	10-40
Creatinine (µmol/L)	81	56	51	62-106
BUN (mmol/L)	4.2	4.3	4.2	2.5-6.4

## Discussion

Our case exemplifies the severity of NF, an uncommon yet devastating infection affecting 0.4-1.99 individuals per 100,000 population globally, with incidence trending upward [10]. Predisposing factors encompass traumatic injuries, thermal burns, arthropod bites, and systemic conditions, including diabetes, malignancy, and renal dysfunction [10]. While NF affects all demographics, elderly patients show increased susceptibility [10]. Approximately half of the cases involve lower extremities, with one-third affecting the upper limbs [10].

The infection commonly originates from skin breach (50%) or blunt trauma (25%), with 70% of affected individuals having pre-existing medical conditions [10,11]. The early clinical manifestations, including localized pain, edema, erythema, and tenderness, often mimic benign soft tissue infections, creating diagnostic challenges (Tables 2, 3) [10,11].

**Table 2:** Symptoms and signs of necrotizing soft tissue infection This table demonstrates the frequency of various clinical signs in patients presenting with necrotizing soft tissue infections. Note that nonspecific signs are more common than classic signs at initial presentation

Sign	Percentage of patients at presentation
Classic	
Bullae	25.6
Skin necrosis	24.1
Crepitus	20.3
Gas on radiographic evaluation	24.8
Nonspecific	
Swelling	80.8
Pain or tenderness	79
Erythema	70.7
Warmth	44
Fever >37.5	40
Hypotension	21.1

**Table 3:** Differential diagnoses and distinguishing features from NSTI NSTI: necrotizing soft tissue infection This table helps clinicians differentiate NSTI from other conditions that may present with similar symptoms

Differential diagnosis	Distinguishing features from NSTI
Cellulitis	1-Often not associated with hemodynamic instability, shock, or severe pain. 2-Less associated with violaceous skin changes
Pyoderma gangrenosum	1-Not associated with sepsis, fever. 2-Associated with inflammatory bowel disease. 3-Progresses slower than NSTI. 3-Violaceous ulcer edges. 4-Resistant to blunt dissection. 5-Typically has negative blood and tissue cultures
Pyomyositis	1-Characterized by abscess formation in muscles. 2-Imaging shows focal muscle swelling and well-demarcated areas of fluid
Deep vein thrombosis	1-Pain is less severe than that of NSTI. 2-Fever may be present, though less common than in NSTI
Erysipelas	1-Raised, sharply demarcated borders

Specific NF variants, including Ludwig's angina (submandibular involvement) and Fournier's gangrene (genital/perineal necrosis), present in characteristic anatomical distributions, often demonstrating fulminant onset with rapid clinical decline [11].

Currently, no single laboratory test definitively establishes NF diagnosis. Retrospective analyses suggest elevated lactate, hyponatremia, leukocytosis, and renal dysfunction correlate with adverse outcomes [7]. Disease-specific scoring tools like LRINEC (Table 4) demonstrate lower predictive accuracy compared to general physiological assessment scores [7].

**Table 4:** Laboratory risk factors for necrotizing fasciitis LRINEC: Laboratory Risk Indicator for Necrotizing Fasciitis The LRINEC score ≥6 has a positive predictive value of 92% and a negative predictive value of 96% for necrotizing fasciitis. Score interpretation: low risk (≤5), moderate risk (6-7), and high risk (≥8)

Variables	Value	Score
White blood count (x10000/µl)	15>	0
	15-25	1+
	25<	2+
Hemoglobin, g/dl	>13.5	0
	11.5-13.5	1+
	11.5>	2+
Sodium, Eq/L	135≤	0
	135>	2+
Glucose, mg/dl	≤180	0
	180<	1+
Creatinine, mg/dl	≤1.6	0
	1.6<	2+
C-reactive protein	mg/dl 15>	0
	≥15mg /dl	4+

A contemporary multicenter investigation introduced the NSTIs (NECROSIS) clinical prediction tool for emergency surgical patients with soft tissue infections [12]. Through comprehensive evaluation of demographics, physiological parameters, laboratory values, examination findings, and imaging results, investigators identified three independent NSTI predictors: hypotension (systolic BP ≤120 mmHg), violaceous skin discoloration, and leukocytosis (>15,000/μL) [12]. When all three factors were present, both specificity and positive predictive value reached 100% in the derivation and validation populations [12].

Achieving source control through prompt and extensive surgical debridement remains the primary factor influencing outcomes in NSTIs [13]. Early surgical intervention dramatically reduces mortality compared to delayed procedures, even when postponed by mere hours [13]. Upon NF diagnosis, immediate surgical intervention is mandatory, with complete excision of all infected tissue being the therapeutic goal [13]. When further debridement is required, patients must return expeditiously to the operating theater [14]. Standard practice involves re-exploration within 12-24 hours of initial surgery [14]. Literature reports indicate NF patients may require 5-40 surgical procedures, including debridements and reconstructive operations [14]. Surgeons should excise tissue with generous margins rather than risk leaving residual infection, thereby reducing recurrence risk [14]. Definitive wound closure with skin grafting occurs only after establishing clean, granulating tissue beds [14].

Medical management

All patients with suspected NF require immediate broad-spectrum antimicrobials following blood culture collection [15]. Initial therapy must provide coverage against Gram-positive, Gram-negative, and anaerobic pathogens [15]. Table 5 outlines guideline-recommended empirical regimens [15]. For hemodynamically unstable patients exhibiting toxic shock features, clindamycin addition helps reduce bacterial toxin production [15]. Immunocompromised individuals warrant empirical antifungal coverage consideration [16].

**Table 5:** Empirical antibiotics for necrotizing soft tissue infections in patients with normal kidney function Doses are for patients with normal kidney function. Adjustment required for renal impairment. Clindamycin is included for its antitoxin effects, particularly important in streptococcal toxic shock syndrome

Patient status	Antibiotic combination
Stable	One of the following antibiotics: · amoxicillin/clavulanate, 1.2/2.2 g every 8 h · ceftriaxone, 2 g every 24 h + metronidazole, 500 mg every 8 h · cefotaxime, 2 g every 8 h + metronidazole, 500 mg every 8 h plus · clindamycin, 600-900 mg every 8 h
Unstable	One of the following antibiotics: · piperacillin/tazobactam, 4.5 g every 6 h · meropenem, 1 g every 8 h · imipenem/cilastatin, 500 mg every 6 h plus one of the following antibiotics: · vancomycin, 25-30 mg/kg loading dose, then 15-20 mg/kg dose every 8 h · daptomycin, 6-8 mg/kg every 24 h · telavancin, 10 mg/kg every 24 h plus · clindamycin, 600-900 mg every 8 h

Adjuncts

Supplementary treatments for NF include hyperbaric oxygen therapy (HBOT) and IVIG administration. Evidence supporting HBOT use comes primarily from observational studies, which may contain selection bias as critically ill patients often cannot receive HBOT due to clinical instability or resource limitations [17]. Two comprehensive meta-analyses examining nearly 50,000 NSTI patients demonstrated reduced mortality with HBOT without affecting amputation rates [17]. HBOT must not delay surgical intervention or compromise intensive care management [17]. While protocols vary institutionally, recommendations typically include 1-2 sessions within 24 hours postdebridement, continuing daily until necrosis resolution [17].

IVIG treatment primarily functions by improving bacterial opsonization and neutralizing bacterial toxins [18]. Notably, NSTI patients frequently exhibit low antibody levels against streptococcal virulence factors, including pyrogenic exotoxin B and superantigens, which IVIG can supplement [18]. Available evidence regarding IVIG efficacy remains inconsistent. Despite lacking randomized controlled trial support, observational studies suggest mortality reduction with IVIG versus antibiotics alone. While optimal dosing remains undefined, high-dose regimens (2 g/kg) are generally accepted [19]. A meta-analysis examining IVIG effectiveness in clindamycin-treated streptococcal toxic shock patients demonstrated 50% mortality reduction [19].

A multicenter prospective investigation of streptococcal NSTI patients showed improved 90-day survival with IVIG use [20]. However, results may reflect shorter surgical intervention times [20]. The INSTINCT randomized trial found no significant outcome differences between IVIG and placebo [20].

## Conclusions

This case highlights the critical importance of maintaining a high index of suspicion for NF, even in seemingly low-risk individuals presenting with what appears to be a simple soft tissue infection. The rapid progression of NF, as demonstrated in our young, previously healthy patient, highlights the potential for devastating outcomes if diagnosis and treatment are delayed. Early recognition, aggressive surgical debridement, appropriate antibiotic therapy, and supportive critical care are paramount in improving patient survival and functional outcomes. While the role of adjunctive therapies such as IVIG and HBOT continues to be debated, their consideration in severe cases, particularly those complicated by TSS, may be warranted.
